# Current smoking is associated with a larger waist circumference and a more androgenic profile in young healthy women from high-risk breast cancer families

**DOI:** 10.1007/s10552-017-0999-3

**Published:** 2018-01-03

**Authors:** Carolina Ellberg, Håkan Olsson, Helena Jernström

**Affiliations:** 1 Division of Oncology and Pathology, Department of Clinical Sciences, Lund University and Skåne University Hospital, Lund, Sweden; 2 Division of Cancer Epidemiology, Department of Clinical Sciences, Lund University and Skåne University Hospital, Lund, Sweden

**Keywords:** Smoking, Testosterone, Oral contraceptives, Androstenedione, Waist-to-hip ratio, Breast cancer

## Abstract

The purpose was to elucidate the interplay between current smoking, anthropometric measurements, and endogenous hormone levels in women ≤ 40 years. Questionnaires on lifestyle and reproductive factors were completed by 269 healthy women from high-risk breast cancer families between 1996 and 2006 in Sweden. Blood samples for analyses of plasma testosterone, estradiol, androstenedione, sex hormone-binding globulin, and body measurements were obtained 5–10 days before predicted onset of the next menstrual period. Women without smoking status, who were currently breastfeeding, or using hormonal contraception other than combined oral contraceptives (OCs) were excluded (*n* = 27). Current smokers (*n* = 57) had larger waist circumference (_adj_*p* = 0.004) and waist-to-hip ratio (WHR) (_adj_*p* = 0.007) than non-smokers (*n* = 185). In non-OC users, adjusted mean androstenedione levels were higher in current smokers compared with non-smokers (10.3 vs. 8.6 nmol/L; _adj_*p* = 0.0002). While in current OC users estradiol levels were higher in smokers compared with non-smokers (22.5 vs. 17.4 pg/mL; _adj_*p* = 0.012). In multivariable models, WHR was associated with both current smoking (_adj_*p* ≤ 0.016) and higher levels of androstenedione (_adj_*p* = 0.05) or bioavailable testosterone (_adj_*p* = 0.001). Among non-OC users, a more androgenic profile was observed in current smokers compared with non-smokers, but not in current OC users. Irrespective of OC use, current smoking was associated with increased waist circumference.

**Electronic supplementary material:**

The online version of this article (10.1007/s10552-017-0999-3) contains supplementary material, which is available to authorized users.

## Introduction

Cigarette smoke is considered a carcinogen, which causes lung cancer as well as several other types of cancer, including pancreatic, liver, ovarian, cervical, and uterine cancers [1]. Whether cigarette smoke causes breast cancer has been heavily debated [2, 3]. Evidence is suggestive of a causal link in which cigarette smoke influences both breast cancer risk and prognosis [4, 5]. However, the underlying mechanisms need to be better elucidated. Early oral contraceptive (OC) use is more common among smokers than among non-smokers [6] and may confound the association between smoking and breast cancer risk. Cigarette smoke contains more than 7,000 chemicals, of which 69 are established carcinogens [1]. Furthermore, smoke also acts as an aromatase inhibitor, which may impact the levels of key hormones for breast tissue due to its role in androgen-to-estrogen conversion [1].

An animal study in female baboons showed that administering nicotine at doses equivalent to that of an average smoker inhibited aromatase function by close to 50% [7, 8]. Studies in postmenopausal women have indicated increased testosterone levels in current smokers compared with non-smokers [9]. Further, estradiol, testosterone, and sex hormone-binding globulin (SHBG) levels increased with increasing cigarette smoke exposure [9, 10]. Similarly, there are studies that indicate an increase in androstenedione in current smokers [2, 10–15].

In a recent study of breast cancer patients, current smokers, compared with non-smokers, were younger, had a lower body weight and body mass index (BMI), had smaller total breast volume, and had an increased waist-to-hip ratio (WHR). Among aromatase inhibitor-treated patients, current smokers had a threefold increased risk of early recurrence compared with non-smokers [16].

Even though smokers often have lower body weight compared with non-smokers, heavy smoking exposure has been associated with weight gain [17]. Despite the lower weight in light to moderate smokers, there is an increased resistance to insulin in smokers, which might seem counterintuitive [17, 18]. This finding may partly be explained by the fact that smoking is associated with an increase in visceral adipose tissue and WHR [17], even when BMI remains unchanged [19]. Women from high-risk breast cancer families were more likely to have gained weight as of age 20 years and to have a WHR > 0.85 compared with controls. Further women from high-risk breast cancer families were more likely to have given up smoking [20].

Because breast cancer develops long before the tumor is clinically detectable, the purpose of this study was to elucidate the interplay between current smoking and anthropometric measurements, as well as their relationship with endogenous hormone levels in young healthy women, with or without current OC use, at a time point when breast cancer might be initiated.

## Materials and methods

### Study population

Between 1996 and 2006, 269 healthy women ≤ 40 years old were included in a study of the impact of lifestyle factors on body constitution and hormone levels, as previously described [21–25]. Potential participants were identified from pedigrees and patient charts from high-risk breast cancer families at the Oncogenetic Clinic at Skånes University Hospital, Lund. For families where the person who had been to the Oncogenetic Clinic was not eligible, he or she was asked whether they would be willing to inform relatives of the study. The criteria for being considered belonging to a high-risk breast cancer family if one out of three cases in a family was diagnosed with breast cancer before age 50, if one out of two cases of breast cancer was diagnosed before age 40, one case of breast cancer was diagnosed before age 30, had a male relative with a breast cancer diagnosis, or if there was ovarian cancer diagnoses in the family. To be included, the women had to have no previous prophylactic mastectomies or bilateral oophorectomies and no previous cancer diagnoses. Further, the women had to have menstrual cycles because the study visits occurred during cycle days 5–10, as well as 5–10 days prior to the predicted onset of the next menstrual cycle (i.e., days 18–23 in most women). During these visits, a trained research nurse obtained blood samples and body measurements including height, weight, waist and hip circumferences, and breast volume. Breast volumes were calculated as approximated pyramids (base area × height/3), and measurements were taken when the women were standing on hands and knees. Blood samples were collected between 7:15 am and 12:15 pm. Participants filled out questionnaires on lifestyle and reproductive factors including current and former smoking as well as history of hormonal contraceptive use .

Out of the 269 women in this cohort, 86 (36%) women belonged to families with a known deleterious *BRCA1* mutation, 22 (9%) belonged to families with a known deleterious *BRCA2* mutation, 103 (43%) belonged to a family where no *BRCA1*/*2* mutation was identified, and 30 (12%) of the women belonged to families where no mutation screening had been carried out. All women signed a written informed consent and the Lund University Ethics Committee approved the study.

### Laboratory methods

Laboratory methods for all hormones have been previously described in detail [23]. Testosterone (T) [23], estradiol (E2) [25], and SHBG [25] in EDTA-plasma were measured using electrochemiluminescent immunoassay by an Elecsys 1010/2010 Modular analytics E170 analyzer with a Roche Elecsys 1010/2010 (Roche Diagnostics, Mannheim, Germany). The intra-assay variation was 2.5–6.8% for testosterone, 1.9–5.7% for estradiol, and 1.8–4.0% for SHBG. The limit of detection was 0.069 nmol/L for testosterone, 5 pg/mL for estradiol, and 0.35 nmol/L for SHBG. Androstenedione (4-androsten-3,17-dione) [23] in EDTA-plasma was analyzed with a Coat-A-Count Direct Androstenedione radioimmunoassay in vitro diagnostic test kit (DPC Skafte, Mölndal, Sweden). The maximum allowed variation was 10% for androstenedione, and the limit of detection was 0.2 nmol/L.

For this study, testosterone (nmol/L) and estradiol (pmol/L) levels were converted to gravimetric units, pg/dL and ng/L, respectively, using a conversion factor of 0.0347 for testosterone and 3.67 for estradiol [26]. The relationship between estradiol and testosterone was investigated using the ratio between the two, which was calculated using the following formula:

$$\frac{{{\text{E}}2}}{{\text{T}}}=\frac{{{\text{E}}2\left( {{\text{pg}}/{\text{mL}}} \right)}}{{{\text{T}}\left( {{\text{ng}}/{\text{dL}}} \right)}} \times 10.$$

SHBG binds testosterone with high affinity. The remaining testosterone levels are, to some extent, bound by albumin, but with low affinity. The T/SHBG molar ratio was used as a proxy for bioavailable testosterone [27].

### Statistics

All analyses were conducted using IBM SPSS statistics version 22.0 [28]. Weight, BMI, breast volume, waist circumference, and plasma hormone levels were not normally distributed and were transformed using the natural logarithm (Ln) to obtain a more normal distribution. Current OC use and smoking status were used as categorical variables. An interaction term between current OC use and current smoking was calculated in order to assess potential interactions between these exposures on the outcome variables.

Generalized linear models were used to obtain adjusted geometric means with 95% Wald Confidence Intervals (CI) via estimated marginal means for anthropometric factors and hormonal levels in current smokers and non-smokers. All models investigating anthropometric factors in relation to current smoking status were adjusted for age, nulliparity, and current OC use. Further, depending on the outcome variable, the models for waist circumference, hip circumference, and WHR were also adjusted for weight and height, the model for weight was also adjusted for WHR and height, the models for height and total breast volume were also adjusted for WHR and weight, and the model for BMI was also adjusted for WHR. A *p* value < 0.05 was considered as statistically significant. Nominal *p* values without adjustment for multiple testing are presented.

## Results

**Fig. 1:**
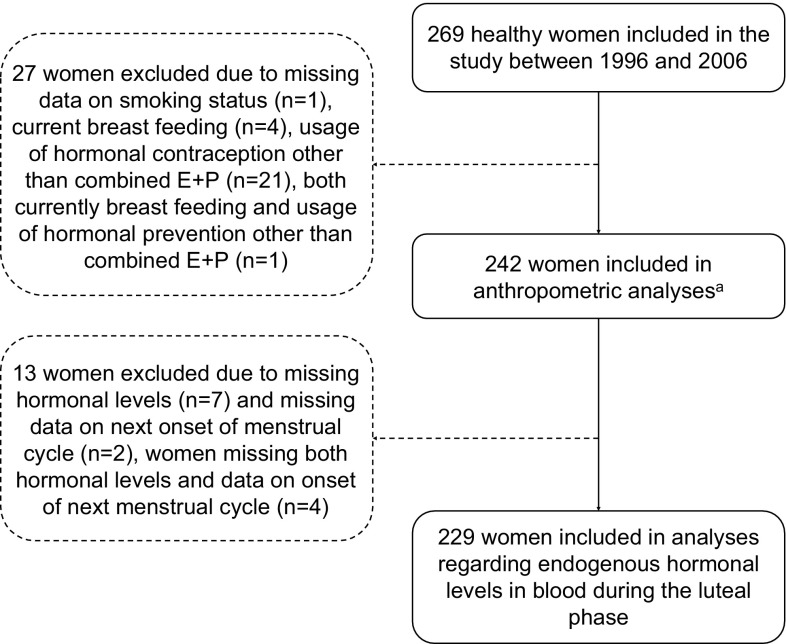
Flowchart of the inclusion and exclusion criteria for the study. **a** In analyses of breast volume, two women were excluded due to missing data on breast volume and seven women were excluded due to previous breast operations, leaving 233 included in the analyses

The characteristics of the study population are presented in Table 1. Due to exclusion described in the flowchart in Fig. 1, analyses of the impacts of current smoking on anthropometric factors or hormone levels were conducted for 242 and 229 women, respectively. Included and excluded women were similar with respect to anthropometric and lifestyle factors, and a comparison of included and excluded women is presented in Supplementary Table 1. The median age at inclusion of the 242 women was 29 years. 46% were parous and 38% were current OC users, whereas 92% had ever used OCs. 24% were current smokers, but 42% reported smoking cigarettes at some point. In women who reported having ever smoked, the median start age was 15 years. Non-smokers contained both former smokers and never smokers, and characteristics for the three groups are displayed in Table 1 for the 242 and 229 women included in the multivariable analyses. Former smokers were slightly older and were more often parous compared with never smokers. Current smokers started smoking at a younger age compared with former smokers, and current and former smokers had a higher frequency of ever using OCs compared with never smokers.

**Table 1:** Reproductive and anthropometric characteristics by smoking status among women from high-risk breast cancer families, *n* = 242

	All women (*n* = 242)		Never smokers (*n* = 140)		Former smokers (*n* = 45)		Current smokers (*n* = 57)	
	Median or *n* (%)	IQR	Median or *n* (%)	IQR	Median or *n* (%)	IQR	Median or *n* (%)	IQR
Age at inclusion, years	29	24–35	29	23–34	31	28–37	29	25–35
Year of birth	1970	1964–1976	1971	1965–1978	1967	1964–1972	1969	1963–1974
Age at menarche	13^a^	12–14	13^a^	12–14	13	12–13	12	12–13
Parous, yes	112 (46)		60 (43)		26 (58)		26 (46)	
Weight, kg	64	58–74	63	57–73	66	60–75	65	59–74
Height, cm	168	164–172	168	164–172	168	165–172	167	164–172
Body mass index, BMI	23	21–25	22	20–25	23	21–26	23	21–26
Waist circumference, cm	76	70–83	75	69–82	74	71–82	78	72–84
Hip circumference, cm	101	95–106	100	94–105	102	95–106	101	95–106
Waist-to-hip ratio, WHR	0.76	0.73–0.80	0.75	0.72–0.79	0.76	0.73–0.78	0.78	0.74–0.82
Total breast volume, cm^3^	756^b^	546–1,101	695^b^	493–1,001	748	557–1,194	816	613–1,195
Start age for ever OC use	17	16–18	18	16–19	16	15–18	16	15–17
Ever use of OC, yes	222 (92)		123 (88)		43 (96)		56 (98)	
Current use of OC, yes	91 (38)		51 (36)		17 (38)		23 (40)	
Start age for ever smokers	15^a^	14–18			17^a^	14–19	15	14–17
Time since smoking, years					4	1–10		

^a^Missing data for one woman

^b^Missing data for two women

### Current smoking status in relation to anthropometric measures

The impact of current smoking on anthropometric factors was assessed. Since there was no effect modification from current OC use on the association between current smoking and any of the anthropometric factors, no stratification according to current OC use was performed. Anthropometric factors in current smokers and non-smokers are presented in Table 2. Compared with non-smokers, current smokers had a significantly larger adjusted mean waist circumference (78.2 vs. 76.5 cm; _adj_*p* = 0.004) and WHR (0.78 vs. 0.76; _adj_*p* = 0.007). No differences between current smokers and non-smokers were observed for hip circumference (_adj_*p* = 0.47), weight (_adj_*p* = 0.85), height (_adj_*p* = 0.95), BMI (_adj_*p* = 0.86), or total breast volume (_adj_*p* = 0.83).

**Table 2:** Crude and adjusted geometric means and 95% CI for anthropometric factors in current smokers and non-smokers, *n* = 242

	*n*	Crude geometric mean	95% Wald CI			Adj geometric mean	95% Wald CI		
			Lower	Upper	*p* value		Lower	Upper	Adj *p* value
Waist circumference^a,b,c^ (cm)									
Current smoker	57	79.8	76.7	82.3	0.022	78.2	77.2	79.2	0.004
Non-smoker	185	75.9	75.2	77.5		76.5	75.9	77.1	
Hip circumference^a,b,c^ (cm)									
Current smoker	57	101.8	99.6	104.1	0.36	100.7	99.9	101.5	0.47
Non-smoker	185	100.6	99.4	101.9		101.0	100.6	101.5	
WHR^a,b,c^									
Current smoker	57	0.79	0.77	0.80	0.004	0.78	0.77	0.79	0.007
Non-smoker	185	0.76	0.75	0.77		0.76	0.75	0.77	
Weight^a,c,d^ (kg)									
Current smoker	57	67.7	64.6	70.9	0.21	66.0	63.7	68.5	0.85
Non-smoker	185	65.4	63.7	67.1		66.3	64.9	67.7	
Height^a,b,d^ (cm)									
Current smoker	57	168.2	166.6	169.8	0.97	168.1	166.6	169.5	0.95
Non-smoker	185	168.2	167.3	169.0		168.1	167.3	169.9	
BMI^a,d^ (kg/m^2^)									
Current smoker	57	24.0	22.9	25.0	0.17	23.4	22.5	24.2	0.86
Non-smoker	185	23.1	22.6	23.7		23.5	23.0	24.0	
Total breast volume^a,b,c,d^ (cm^3^)									
Current smoker	54	818	708	946	0.18	741	670	818	0.83
Non-smoker	179	731	675	792		750	709	793	

Adjusted for ^a^age, current OC use, and nulliparity, ^b^weight, ^c^height, ^d^WHR

### Current smoking status in relation to endogenous hormone levels

Because OCs are known to influence hormone levels, a formal interaction analysis between current OC use and current smoking for each hormone was performed. There were significant interactions between current smoking and current OC use with respect to estradiol levels (*p*_int _= 0.025) and the E2/T ratio (*p*_int_ = 0.024), but not for the other hormones. Women were stratified according to current OC use. Hormone levels in non-OC users in relation to current smoking are presented in Table 3. Compared with non-smokers, current smokers had higher adjusted mean levels of androstenedione (_adj_*p* = 0.0002) and total testosterone (_adj_*p* = 0.048), as well as a lower E2/T ratio (_adj_*p* = 0.027). No differences between non-smokers and current smokers were observed for bioavailable testosterone, SHBG, or estradiol. Hormone levels in current OC users are presented in Table 4. Current smokers had significantly higher adjusted mean estradiol levels compared with non-smokers (_adj_*p* = 0.012). None of the other hormones differed according to current smoking status in OC users.

**Table 3:** Crude and adjusted geometric means and 95% CI for hormone levels in non-OC users for current smokers and non-smokers, *n* = 142

	*n*	Crude geometric mean	95% Wald CI			Adj geometric mean	95% Wald CI		
			Lower	Upper	*p* value		Lower	Upper	Adj *p* value
Androstenedione (nmol/L)									
Current smoker	32	10.2	9.2	11.3	0.004	10.3	9.5	11.2	0.0002
Non-smoker	110	8.6	8.2	9.1		8.6	8.3	9.0	
Total testosterone (ng/dL)									
Current smoker	32	49.6	43.1	57.1	0.14	50.9	45.1	57.6	0.048
Non-smoker	110	44.0	40.8	47.5		44.2	41.3	47.3	
T/SHBG molar ratio									
Current smoker	32	0.076	0.061	0.094	0.21	0.074	0.063	0.088	0.20
Non-smoker	110	0.065	0.057	0.072		0.065	0.060	0.072	
SHBG (nmol/L)									
Current smoker	32	22.8	19.6	26.5	0.65	23.8	21.0	27.0	0.82
Non-smoker	110	23.7	21.8	25.7		23.4	21.9	25.1	
Estradiol (pg/mL)									
Current smoker	32	101.7	84.9	121.9	0.23	100.8	83.9	121.0	0.24
Non-smoker	110	115.5	104.7	127.3		114.1	103.1	126.2	
E2/T ratio									
Current smoker	32	20.5	16.6	25.3	0.045	19.8	16.1	24.3	0.027
Non-smoker	110	26.2	23.4	29.4		25.8	23.0	28.9	

Adjusted for age, nulliparity, weight, height, WHR, and days until the next menstrual period

**Table 4:** Crude and adjusted geometric means and 95% CI for hormone levels in current OC users for current smokers and non-smokers, *n* = 87

	*n*	Crude geometric mean	95% Wald CI			Adj geometric mean	95% Wald CI		
			Lower	Upper	*p* value		Lower	Upper	Adj *p* value
Androstenedione (nmol/L)									
Current smoker	21	6.6	5.7	7.8	0.13	5.9	5.0	6.9	0.25
Non-smoker	66	5.8	5.3	6.3		5.3	4.8	5.9	
Total testosterone (ng/dL)									
Current smoker	21	31.4	26.0	37.9	0.39	28.6	23.5	34.9	0.52
Non-smoker	66	28.5	25.6	31.7		26.8	23.7	30.3	
T/SHBG molar ratio									
Current smoker	21	0.016	0.012	0.021	0.35	0.016	0.012	0.021	0.39
Non-smoker	66	0.014	0.012	0.016		0.014	0.011	0.016	
SHBG (nmol/L)									
Current smoker	21	66.8	54.2	82.3	0.66	63.4	51.6	78.0	0.56
Non-smoker	66	70.6	62.7	79.4		67.7	59.5	77.0	
Estradiol (pg/mL)									
Current smoker	21	22.2	18.5	26.6	0.027	22.5	18.7	27.1	0.012
Non-smoker	66	17.5	15.8	19.4		17.4	15.5	19.6	
E2/T ratio									
Current smoker	21	7.1	5.7	8.8	0.27	7.9	6.2	9.9	0.14
Non-smoker	66	6.1	5.4	6.9		6.5	5.6	7.5	

Adjusted for age, nulliparity, weight, height, WHR, and days until the next menstrual period

### Impacts of current smoking and hormone levels on WHR

There was no effect modification between current smoking and current OC use on WHR. The following mutually adjusted multivariable models investigating the impact of current smoking and hormone levels on WHR were therefore conducted for current OC users and non-OC users combined (*n* = 229). The models were adjusted for age, nulliparity, weight, height, current OC use, and days until the next menstrual period. One hormone per calculation was included because of the high correlation between the measured hormones. These models are presented in Table 5. Current smoking was positively associated with a larger WHR in all models. However, only higher levels of androstenedione (_adj_*p* = 0.050) and bioavailable testosterone (_adj_*p* = 0.001) and lower levels of SHBG (_adj_*p* < 0.0001) were significantly associated with larger WHR.

**Table 5:** Mutually adjusted multivariable models of the impacts of current smoking and hormone levels on Ln-transformed WHR, *n* = 229

	*β*	95% Wald CI		*p* value
		Lower	Upper	
Intercept	0.214	0.023	0.405	0.028
Androstenedione Ln(nmol/L)	0.021	− 0.000013	0.043	0.050
Current smoker, yes	0.018	0.003	0.033	0.016
Weight LN(kg)	0.183	0.143	0.224	< 0.0001
Height (cm)	− 0.002	− 0.003	− 0.001	0.003
Intercept	0.234	0.038	0.430	0.019
Total testosterone Ln(ng/dL)	0.004	− 0.012	0.019	0.662
Current smoker, yes	0.021	0.007	0.036	0.004
Weight Ln(kg)	0.195	0.156	0.235	< 0.0001
Height (cm)	− 0.002	− 0.003	− 0.001	0.001
Intercept	0.314	0.125	0.504	0.001
T/SHBG molar ratio	0.018	0.008	0.028	0.001
Current smoker, yes	0.019	0.005	0.033	0.009
Weight Ln(kg)	0.171	0.131	0.212	< 0.0001
Height (cm)	− 0.001	− 0.003	− 0.0004	0.009
Intercept	0.345	0.160	0.539	0.0003
SHBG Ln(nmol/L)	− 0.029	− 0.043	− 0.016	< 0.0001
Current smoker, yes	0.020	− 0.00	0.034	0.004
Weight Ln(kg)	0.174	0.135	0.212	< 0.0001
Height (cm)	− 0.002	− 0.003	− 0.0004	0.007
Intercept	0.255	0.056	0.454	0.012
E2 Ln(pg/mL)	− 0.002	− 0.014	0.010	0.749
Current smoker, yes	0.021	0.007	0.036	0.004
Weight Ln(kg)	0.198	0.160	0.237	< 0.0001
Height (cm)	− 0.002	− 0.003	− 0.001	0.001
Intercept	0.258	0.0063	0.452	0.009
E2/T ratio	− 0.00	− 0.014	0.008	0.563
Current smoker, yes	0.021	0.007	0.036	0.004
Weight Ln(kg)	0.197	0.158	0.235	< 0.0001
Height (cm)	− 0.002	− 0.003	− 0.001	0.001

Additionally, all models were adjusted for age, nulliparity, current OC use, and days until the next menstrual period

## Discussion

The main finding of this study was that current smoking was associated with a more androgenic profile in non-OC users, and larger waist circumference and larger WHR irrespective of current OC status. However, in current OC users, estradiol levels were higher in current smokers compared with non-smokers. Even though current smokers had significantly larger waist circumference and higher WHR, they had similar BMI and breast volume compared with non-smokers.

It needs to be mentioned that this population was generally slim, so, despite the increase in WHR or waist circumference in smokers, most women had a WHR within the recommended WHO limits. However, in a recent study by Iyengar et al. [29], even small changes in body constitution in a normal weight breast cancer high-risk population can be related to the presence of breast white adipose tissue inflammation. Metabolically, these women present with an obese phenotype in terms of inflammation and aromatase activity, despite being within the recommended limits of BMI [29]. The observed difference in body fat distribution towards more abdominal fat in relation to androstenedione and testosterone suggests that current smoking in the present cohort was associated with a more inflammatory and/or androgenic profile at the age when breast cancer is likely to be initiated.

It has been suggested that smoking may induce hyperandrogenism in premenopausal women [30], and that it may increase the levels of testosterone and androstenedione in postmenopausal women [2]. Both testosterone and androstenedione levels have been implicated as breast cancer risk factors in pre- and postmenopausal women [1, 2, 15, 31, 32]. In the present study, increased androstenedione and testosterone levels were observed in current smokers who were non-OC users, indicating that current smoking might be a contributing factor to the increase in androgens. Nicotine also acts as an aromatase inhibitor, which may partly explain the androgenic profile [2]. Furthermore, this increase in androgen levels might be related to increasing WHR. With regard to the increased estradiol levels in current smokers using OCs, we hypothesized that there were two plausible explanations: one explanation is that there is reduced efficacy of OCs while currently smoking [33]; the other explanation is lower adherence to taking the pill because smokers have been reported to have lower adherence to taking medication [34, 35].

With regard to baseline characteristics, the included and excluded women were similar, suggesting that the exclusion of some women was unlikely to introduce bias. The prevalence of smokers in the study population used in the present study resembles the population with regard to the number of smokers [36], and the prevalence of smokers and ever smokers was similar in women who were excluded due to missing data on hormone levels or anthropometric measures. Moreover, all anthropometric measures used in the present study were obtained by a trained nurse. The present study is somewhat limited with regard to population size, which limits the possibility for stratification, such as by dose of cigarettes per day.

The group of non-smokers includes both never smokers and former smokers. These groups were slightly different with respect to age, parity, and BMI. The main reason for combining former and never smokers was that the aim of the study was to elucidate the impact of current smoking in young women on hormone levels and anthropometrics. The slightly higher age along with the higher parity among former smokers compared with never smokers is most likely explained by women quitting smoking before or when getting pregnant. Analyses were performed with stratification according to smoking status (as in never, ever, and former smokers) and the results remained essentially the same (data not shown). The women all came from high-risk families, and analyses were also performed after stratification based on mutation status to make sure that results were not associated with mutations in the *BRCA1*/*2* genes, but it did not alter the results (data not shown).

Regarding hormone levels, if more sensitive methods had been available at the time of analyses, such as those available today, it is possible that the results among current OC users with respect to hormone levels in current smokers and non-smokers could have been different because several of the current OC users had low hormone levels. However, the findings are similar to other observations [2] and it is therefore unlikely that this issue had a significant impact on our results.

Taken together, we have observed associations between current smoking and different risk factors for breast cancer in young healthy women from high-risk breast cancer families at the age when breast cancer might be initiated. Current smoking was associated with increased WHR, which is a risk factor for increased inflammation or even a metabolically obese phenotype [17], and increased levels of androgens such as testosterone and androstenedione, which are implicated as risk factors for breast cancer and hyperandrogenism [32]. We propose that smoking needs to be taken into account when studying the relationship between these factors. Because many of the *BRCA1*/*2* mutation carriers in our cohort had undergone prophylactic surgery after inclusion in the study, there were too few cases of breast cancer for a meaningful comparison of the breast cancer risk between current smokers and non-smokers. Breast cancer risk in relation to smoking, OC use, anthropometric factors, and hormone levels needs to be studied in another substantially larger cohort.

## Conclusion

In conclusion, we found that among non-OC users, current smokers had more androgenic profile, mainly driven by increased androstenedione levels, compared with non-smokers. In current OC users, higher estradiol levels were found among current smokers compared with non-smokers. Irrespective of OC use, current smoking was associated with increased waist circumference. History of OC use should be considered in studies of smoking in women.

## Electronic supplementary material

Below is the link to the electronic supplementary material.

Supplementary material 1 (XLSX 16 KB)
